# 1190. Effect of Pulsed Xenon Ultraviolet Light (PX-UV) on Clonal Recovery of *Escherichia coli* in a Prospective, Sham-controlled, Double-blinded, Interventional, Crossover Trial Conducted in Two Detroit Hospitals

**DOI:** 10.1093/ofid/ofac492.1025

**Published:** 2022-12-15

**Authors:** Thanuri Navarathna, Piyali Chatterjee, Landon Ashby, Hosoon Choi, Munok Hwang, Sorabh Dhar, Keith S Kaye, Chetan Jinadatha

**Affiliations:** Central Texas Veterans Health Care System, Temple, Texas; Central Texas Veterans Health Care System, Temple, Texas; Central Texas Veterans Health Care System, Temple, Texas; Central Texas Veterans Health Care System, Temple, Texas; Central Texas Veterans Health Care System, Temple, Texas; Wayne State University, Detroit, Michigan; Rutgers - Robert Wood Johnson Medical School, New Brunswick, New Jersey; Central Texas Veterans Health Care System, Temple, Texas

## Abstract

**Background:**

Healthcare-associated infections (HAIs) can be caused by some multidrug-resistant *Escherichia coli*, most commonly due to production of an extended spectrum beta-lactamase (ESBL), and lead to increased morbidity and mortality. Pulsed Xenon Ultraviolet light (PX-UV), in combination with terminal cleaning, has been shown to improve disinfection and has the potential to lower HAIs by reducing the horizontal spread of infections in hospitals. Here, we assess the effect of PX-UV on the clonal recovery pattern of several *E. coli* sequence types (STs) using Whole Genome Sequencing (WGS).

**Methods:**

A prospective, sham-controlled, double-blinded, interventional, crossover trial was conducted to compare standard terminal cleaning with PX-UV (intervention, Group Q) and standard terminal cleaning with sham UV (control, Group W) in 2 Detroit hospitals from 2017 to 2020. Treatments lasted 12 months before crossover, with a 6-month washout period in between (Group R) during which UV was not used. A total of 67 *E. coli* samples were collected. WGS of the isolates was performed using the Nextseq 550 (Illumina). After *de novo* assembly, BioNumerics calculation engine (v7.6) was used to complete Whole Genome Multilocus Sequence Type (wgMLST) analysis, assembly free and assembly-based call, and construction of minimum spanning tree (MST).

**Results:**

The total number of different STs found for the intervention UV device group (Q) and the sham UV device group (W) was 6 while the washout group (R) was 5. Out of the 9 total STs obtained, the most common was ST131 (Table 1). In Group Q, 11 of ST131 were found; 15 were found in Group W. During the washout period (R) 17 of ST131 were found. All other STs had 3 or less circulating clusters. After UV treatment ST1193, ST399, and ST7394 were not recovered.

**Fig. 1:**
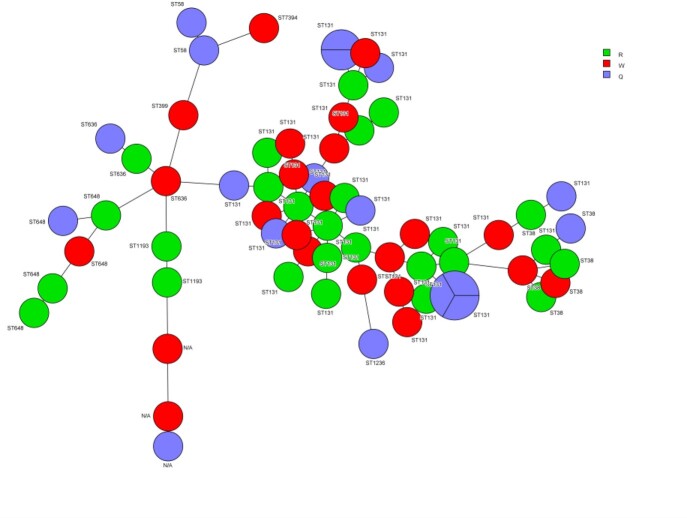
Minimum spanning tree (MST) for E. coli

**Table 1:**
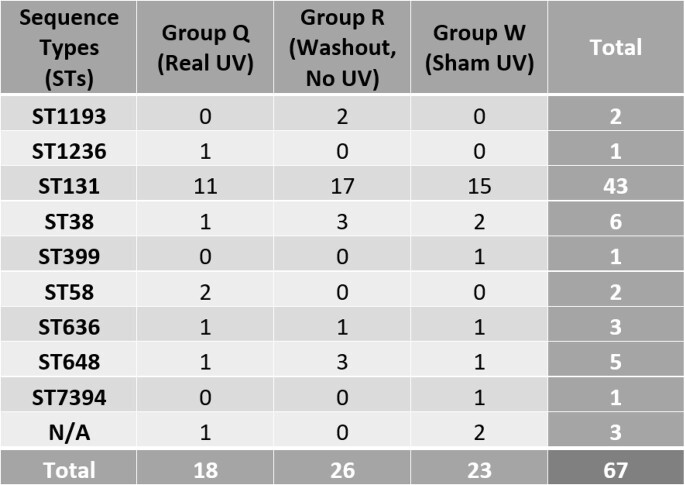
Total number of each sequence type (ST) per treatment group

**Conclusion:**

During UV intervention 3 different STs (ST1193, ST399, ST7394) were not recovered, but there were negligible changes to the frequency of recovery of the other 6 STs as compared to sham UV treatment. ST131 was the dominant *E. coli* ST found in Detroit, which is consistent with previously published data stating ST131 as the predominant strain. While PX-UV has previously demonstrated effectiveness on decreasing bioburden, our data does not indicate any remarkable change in clonality and prevalence of *E. coli* STs after PX-UV use.

**Disclosures:**

**Piyali Chatterjee, PhD**, AHRQ Grant # 1R03HS027667-01: Grant/Research Support|AHRQ Grant # 1R03HS027667-01: Central Texas Veterans Health Care System **Keith S. Kaye, MD, MPH**, Allecra: Advisor/Consultant|GlaxoSmithKline plc.: Receiving symposia honoraria|GlaxoSmithKline plc.: GlaxoSmithKline plc.-sponsored study 212502|Merck: Advisor/Consultant|qpex: Advisor/Consultant|Shionogi: Grant/Research Support|Spero: Advisor/Consultant **Chetan Jinadatha, MD, MPH**, AHRQ R01 Grant-5R01HS025598: Grant/Research Support|EOS Surfaces: Copper Coupons and materials for testing.

